# Findings of transoesophageal echocardiogram in appropriately anticoagulated patients with persistent atrial fibrillation prior to planned cardioversion

**DOI:** 10.1186/s12872-017-0503-8

**Published:** 2017-02-23

**Authors:** Jūratė Barysienė, Aistė Žebrauskaitė, Dovilė Petrikonytė, Germanas Marinskis, Sigita Aidietienė, Audrius Aidietis

**Affiliations:** 1 Centre of Cardiology and Angiology, Vilnius University Hospital Santariskiu Clinics, 2 Santariškių St., LT -08661 Vilnius, Lithuania; 2 Clinic of Cardiovascular Diseases, Faculty of Medicine, Vilnius University, 21 Čiurlionio St., LT-03101 Vilnius, Lithuania

**Keywords:** Atrial fibrillation, Non-vitamin K antagonist oral anticoagulants, Anticoagulation, Cardioversion, Transoesophageal echocardiogram, Thromboembolism

## Abstract

**Background:**

To evaluate a diagnostic value of transoesophageal echocardiogram (TEE) in appropriately anticoagulated patients with a non-valvular atrial fibrillation (AF) and to establish possible additional indications for TEE; to evaluate the incidence of left atrial (LA) thrombi in appropriately anticoagulated patients in daily clinical practice.

**Methods:**

This retrospective study analyses data of 432 patients who had been anticoagulated by means of oral anticoagulants (OACs) prior to planned cardioversion during the period from 2012 to 2015. Thromboembolic (TE) and bleeding risks were assessed using CHA2DS2-VASc and HAS-BLED scores. Transthoracic and transoesophageal echocardiograms were evaluated. TE complications during 30 days after discharge were assessed.

**Results:**

432 patients were selected, aged from 22 to 89 years (mean 65.0 ±11.5), 277 (64.1%) males and 155 (35.9%) females, 306 (70.8%) on warfarin and 126 (29.2%) on non-vitamin K antagonist oral anticoagulants (NOAC). Mean CHA2DS2-VASc score was 3.5 ±1.5. TEE was performed for 120 (27.8%) patients, more frequently for patients on NOACs and for ones with III° LA enlargement.

TEE revealed LA thrombi in seven (5.8%) of the patients. In warfarin and NOACs groups thrombi were revealed in five (7.0%) and two (4.1%) patients, respectively. TEE did not reveal any thrombi in patients with normal left ventricular (LV) function; however, thrombi were found in two (6.1%) patients with slightly decreased LV function, and in five (17.9%) patients with markedly decreased LV function.

In patients with decreased left ventricular ejection fraction (LVEF) thrombi in LA were found more frequently than in patients with normal and slightly decreased LVEF (17.9% vs 2.2%, p=0.008). CHA2DS2-VASc score of all 7 patients was ≥5. None of the patients after cardioversion had TE complications 30 days after discharge.

**Conclusions:**

The risk of LA thrombi in patients prepared for scheduled cardioversion in line with the guidelines is low. Higher risk of thrombi was present in patients with decreased LVEF (≤40%), CHA2DS2-VASc ≥5. In order to assess more accurately indications to perform TEE for appropriately anticoagulated patients prior to scheduled cardioversion a study with larger number of patients is required.

## Background

Atrial fibrillation (AF) is the most common long-lasting supraventricular arrhythmia [1]. In order to improve the condition of patients suffering from symptomatic persistent AF, restoration of sinus rhythm (SR) can be performed.

Ischemic stroke is the most common complication of AF, with the risk remaining high if prophylaxis of thromboembolic (TE) complications is not administered. AF is the cause of every fourth/fifth ischemic stroke and mortality of these strokes is two times higher in comparison with other types of ischemic stroke [1, 2].

Oral anticoagulants (OACs) may reduce the risk of AF related ischemic stroke by 60 – 80%. The risk of TE complications increases during restoration of SR [1]. The incidence of TE events in patients who were not treated with OACs before restoration of SR ranges from 5 to 7% [3], while in patients appropriately prepared for scheduled cardioversion, the incidence of these complications can be decreased to 1.0% [4, 5].

To prevent TE complications before scheduled SR restoration vitamin K antagonists (VKA) [1, 6] or non-vitamin K antagonist oral anticoagulants (NOACs) are administered [1, 6]. Transoesophageal echocardiogram (TEE) for detection of intracardiac thrombi can be performed in order to assess the efficacy of anticoagulation therapy or as an alternative for treatment with OACs [1, 6].

Furthermore, changes of pharmacogenomics of drugs action are more frequent in patients taking VKA. Actually, only two thirds of the patients on warfarin achieve the therapeutic international normalized ratio (INR) interval for  > 64% of period required [7].

Therefore, the duration of preparation for scheduled cardioversion lasts from two to three months [8, 9]. The action of NOACs is stable, dosage is simple, preparation for scheduled SR restoration is shorter. On the other hand, some patients do not comply even with this simplified NOAC administering schedule [10]. This is why physicians have doubts concerning the reliability of anticoagulation before scheduled cardioversion and they perform additional TEE.

The aims of this retrospective study are to evaluate the expedience of TEE before cardioversion in patients properly anticoagulated with OACs suffering from persistent non-valvular AF; to assess the incidence of detection of intracardiac thrombi; to evaluate risk factors of thrombi formation, and to establish additional indications for administering TEE before scheduled SR restoration procedure.

### What’s new?

In patients with atrial fibrillation appropriately prepared with oral anticoagulants for scheduled cardioversion, risk of thromboembolic complications remains below 1.0%, with the incidence of asymptomatic left atrium thrombi up to 7.7% [11, 12]. The aim is to establish clinical criteria of appropriately anticoagulated patients who are at higher risk of thromboembolic complications and for whom transoesophageal echocardiogram would be beneficial before sinus rhythm restoration.

The higher risk of cardiac thrombi is in patients with decreased left ventricular ejection fraction (≤40%), CHA2DS2-VASc ≥ 5.

## Methods

This retrospective study was performed at the Centre of Cardiology and Angiology at Vilnius University Hospital Santariskiu Clinics and included patients who were ≥18 years old, suffering from non-valvular AF and who were prepared for scheduled SR restoration using OACs.

During the period from October 2012 to November 2015, 2940 patients underwent scheduled electrical cardioversion. 2987 patients underwent TEE; this number included 567 patients with persistent non-valvular AF who were prepared for direct current cardioversion (DCC) using OACs; 432 patients were properly anticoagulated and included into this study. The data were obtained from Hospital electronic database, out-patient and in-patient case files. The study was approved by local Bioethics committee.

AF without moderate or high degree stenosis of mitral valve or presence of mechanical valve prosthesis was considered to be non-valvular. Prior to scheduled cardioversion, the patients for ≥3 weeks used NOACs (dabigatran, rivaroxaban, apixaban) or warfarin, maintaining documented INR 2.0-3.0, for not less than 3 subsequent weeks. Individual dosing of NOAC drugs was done according to recommendations. Since it is difficult to confirm directly that patients are indeed taking NOACs properly, we considered to have patients’ verbal confirmation and signature in hospital file confirming that she/he did not miss the dosage.

The following criteria were evaluated: patient age, sex, body mass index (BMI), concomitant diseases (arterial hypertension (AH), diabetes mellitus (DM), heart failure (HF), former stroke or transient ischemic attack (TIA), coronary artery disease (CAD). The risk of ischemic stroke and systemic embolism was evaluated using CHA2DS2-VASc scale [13], the risk of bleeding was assessed using HAS-BLED scale [14].

Left ventricular hypertrophy (LVH), size of left atrium (LA) and left ventricular ejection fraction (LVEF) were assessed using trans-thoracic echocardiography. LVH was diagnosed if interventricular septum and posterior left ventricle (LV) wall thickness equaled or exceeded 11 mm [15]. The size of LA was assessed according to atrial volume in 4-chamber view: no enlargement 22–58 ml, I° enlargement 59–68 ml, II° enlargement 69–78 ml, III° enlargement ≥79 ml [15]. LV systolic function was evaluated by LVEF: normal ≥50%, slightly decreased 41–49%, decreased ≤40% [16]. TEE was performed in 120 patients. TEE was performed more frequently for patients on NOACs because of insufficient confidence and experience of physicians with these drugs, also because it was not possible to check the effect of the drug. The decision to perform TEE was made on the assumption of the physicians that expected risk of TE complications was higher. HF or IIIº LA enlargement, especially their combination, were considered as main factors influencing decision to perform TEE. All patients were studied using Philips iE33 ultrasound machine. All TEE images obtained in each patient were recorded as movie images on a digital media for display and evaluation in real time. TEE images of the LA and left atrium appendage (LAA) were evaluated in the horizontal (0°) plane and in the plane at rotation of the imaging sector (0°–180°) during continuous visualization of the LAA. Thrombus was defined as an echo-dense, well-circumscribed, uniformly consistent mass with a texture different from that of the LAA wall. If thrombi were present, DCC was not performed.

All the study patients for whom scheduled DCC was performed were interviewed by phone 30 days after restoration of SR, in order to reveal TE complications.

Statistical analysis. The data were processed using SPSS 20 and Microsoft Excel software, Pearson’s chi square tests were applied and standard deviations (SD) were calculated for the mean values.

## Results

**Fig. 1:**
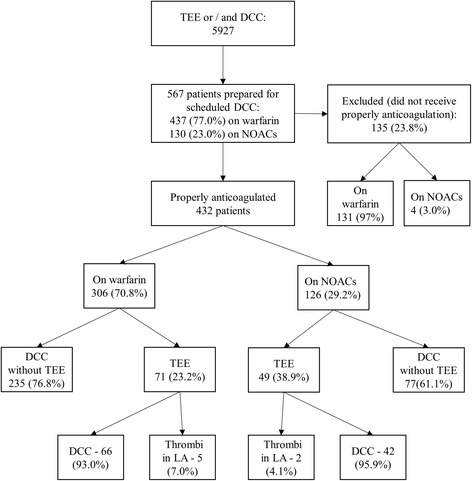
Scheme of patient selection and distribution. DCC – direct current cardioversion, LA – left atrium, NOACs – non-vitamin K antagonist oral anticoagulants, TEE - transoesophageal echocardiography

The total number of the patients with non-valvular AF prepared for scheduled SR restoration using OACs was 567. Appropriate anticoagulation was not achieved in 135 patients and the data of these patients were not included into subsequent analysis. Appropriate anticoagulation for scheduled DCC was achieved in 432 patients and the data of these patients were analyzed. The scheme of patients distribution is shown in Fig. 1.

The age of the patients ranged from 22 to 89 years, mean age was 65.1 ± 11.5 years. There were 277 (64.1%) male and 155 (35.9%) female patients. The mean age of male and female patients was 61.8 ± 11.9 and 70.5 ± 8.1 years, respectively.

The majority of the patients were suffering from AH (92.4%), HF (76.4%) and CAD (50.5%). Decreased LV systolic function was present in 18.3% and LA enlargement was observed in 83.1% of the patients. The distribution of the patients in accordance with demographic data, stroke, TE and bleeding risk, echocardiography findings and concomitant diseases is presented in Table 1.

**Table 1:** Distribution of the patients according to demographic data, echocardiography findings, stroke, thromboembolic and bleeding risk stratification, concomitant diseases

	Patients	Warfarin group	NOACs group
	(n, %)	(n, %)	(n, %)
Male	277 (64.1%)	193 (63.1%)	84 (66.7%)
Female	155 (35.9%)	113 (36.9%)	42 (33.3%)
Age, years ± SD	65.1 ± 11.5	64.6 ± 11.1	64.9 ± 12.5
CHA_2_DS_2_-VASc value ± SD	3.5 ± 1.5	3.6 ± 1.6	3.4 ± 1.5
HAS-BLED value ± SD	0.9 ± 0.9	1.1 ± 0.9	0.9 ± 0.9
HF	330 (76.4%)	238 (77.8%)	92 (73.0%)
AH	399 (92.4%)	280 (91.5%)	119 (94.4%)
CAD	216 (50.0%)	164 (53.6%)	52 (41.3%)
Previous stroke or TIA	23 (5.3%)	21 (6.9%)	2 (1.6%)
DM	54 (12.5%)	41 (13.4%)	13 (10.3%)
BMI 18.5 – 24.9 kg/m^2^	59 (14.5%)	39 (13.5%)	20 (16.8%)
BMI 25.0 – 29.9 kg/m^2^	138 (33.9%)	95 (33.0%)	43 (36.1%)
BMI ≥30 kg/m^2^	209 (51.4%)	153 (53.0%)	56 (47.0%)
LVH	29 (6.7%)	19 (6.2%)	10 (7.9%)
LVEF ≥50%	219 (50.7%)	142 (46.4%)	77 (61.1%)
LVEF 41–49%	134 (31.0%)	99 (32.4%)	35 (27.8%)
LVEF ≤40%	79 (18.3%)	65 (21.2%)	14 (11.1%)
No LA enlargement	73 (16.9%)	41 (13.4%)	32 (25.4%)
I° LA enlargement	126 (29.2%)	91 (29.7%)	35 (27.8%)
II° LA enlargement	126 (29.2%)	94 (30.7%)	32 (25.4%)
III° LA enlargement	107 (24.8%)	80 (26.1%)	27 (21.4%)
Total	432 (100%)	306 (70.8%)	126 (29.2%)

*BMI* body mass index, *CAD* coronary artery disease, *DM* diabetes mellitus, *HF* heart failure, *LA* left atrial, *LVEF* left ventricular ejection fraction, *LVH* left ventricle hypertrophy, *NOACs* non-vitamin K antagonist oral anticoagulants, *SD* standard deviation, *TIA* transient ischemic attack

Warfarin and NOACs were administered for 306 (70.8%) and 126 (29.2%) patients, respectively. In NOACs group 74 (58.7%) patients were on dabigatran, 39 (31.0%) were on rivaroxaban and 13 (10.3) were on apixaban.

Warfarin was administered for the patients in whom the risk of TE complications and bleeding was higher (CHA2DS2-VASc 3.6 (SD ± 1.6) vs 3.4 (SD ± 1.5), *p =* 0.058); HAS-BLED ≥ 3 (5.6% vs 4.8% *p =* 0.08).

For males and elderly (>75 years) patients NOACs were administered more frequently (30.3% vs 27.1%, *p =* 0.27 and 25.4% vs 20.3%, *p =* 0.27, respectively). In patients with good LV systolic function, administration of NOACs was more frequent (61.1% vs 46.4%, p < 0.001) and when LV EF was ≤ 40% administration of warfarin was more common (21.2% vs 11.1%, p < 0.001). When there was no LA enlargement, NOACs were administered more frequently (25.4% vs 13.4%, *p =* 0.008), but if LA was enlarged treatment by warfarin was usually preferred (74.6% vs 86.6%, *p =* 0.008). Patients with the history of stroke or TIA were prepared for restoration of SR with warfarin more frequently (6.9% vs 1.6%, *p =* 0.013). Almost one-third of the patients properly prepared for SR restoration (n = 120, 27.8%) underwent TEE before the procedure. This investigation was more frequently performed for the patients treated with NOACs (n = 49, 38.9% vs n = 71, 23.2%, p < 0.001). Detailed characteristics of the groups of the patients are presented in Table 2.

**Table 2:** Distribution of the patients in accordance with decision to perform TEE, with demographic data, thromboembolic and bleeding risk stratification, echocardiography findings and concomitant disease

	TEE performed (n, %)	TEE not performed (n, %)
Male	77 (64.2%)	200 (64.1%)
Female	43 (35.8%)	112 (35.9%)
On warfarin	71 (23.2%)	235 (76.8%)
On NOACs	49 (38.9%)	77 (61.1%)
Mean age, years ± SD	63.5 ± 11.1	65.2 ± 11.6
CHA_2_DS_2_-VASc value ± SD	3.5 ± 1.5	3.6 ± 1.6
HAS-BLED value ± SD	1.1 ± 1.0	1.0 ± 0.8
HF	98 (81.7%)	232 (74.4%)
AH	113 (94.2%)	288 (91.7%)
CAD	54 (45.0%)	162 (51.9%)
Previous stroke or TIA	8 (6.7%)	15 (4.8%)
DM	16 (13.3%)	38 (12.2%)
BMI <25 kg/m^2^	13 (11.1%)	47 (16.2%)
BMI 25,0 – 29,9 kg/m^2^	47 (40.2%)	91 (31.4%)
BMI ≥30 kg/m^2^	60 (49.6%)	152 (52.4%)
LVH	7 (5.8%)	22 (7.1%)
LVEF ≥50%	59 (49.2%)	160 (51.3%)
LVEF 41-49%	33 (27.5%)	105 (33.7%)
LVEF ≤40%	28 (23.3%)	47 (15.1%)
No LA enlargement	19 (15.8%)	54 (17.3%)
I° LA enlargement	24 (20.0%)	102 (32.7%)
II° LA enlargement	38 (31.7%)	88 (28.2%)
III° LA enlargement	39 (32.5%)	68 (21.8%)
Total	120 (27.8%)	312 (72.2%)

*BMI* body mass index, *CAD* coronary artery disease, *DM* diabetes mellitus, *HF* heart failure, *LA* left atrial, *LVEF* left ventricular ejection fraction, *LVH* left ventricle hypertrophy, *NOACs* non-vitamin K antagonist oral anticoagulants, *SD* standard deviation, *TEE* transoesophageal echocardiography, *TIA* transient ischemic attack

The total score of CHA2DS2-VASc, HAS-BLED and patient age had no influence on decision to perform TEE (see Table 3). TEE was performed more frequently for patients with enlargement of LA III° (32.5% vs 21.8%, *p =* 0.01) and suffering from HF (81.7 vs 74.4%, *p =* 0.068).

**Table 3:** Distribution of patients who had and did not have TEE, in accordance with CHA_2_DS_2_-VASc and HAS-BLED risk scores

	CHA_2_DS_2_-VASc score								
	0	1	2	3	4	5	6	7	8
TEE was performed (n, %)	0 0%	9 7.5%	26 21.7%	23 19.2%	32 26.7%	19 15.8%	8 6.7%	3 2.5%	0 0%
TEE was not performed (n, %)	5 1.6%	21 6.7%	65 20.8%	58 18.6%	74 23.7%	55 17.6%	27 8.7%	6 1.9%	1 0.3%
	HAS-BLED score								
	0	1	2	3	4				
TEE was performed (n, %)	42 35.0%	42 35.0%	26 21.7%	7 5.8%	3 2.5%				
TEE was not performed (n, %)	95 30.4%	140 44.9%	64 20.5%	12 3.8%	1 0.3%				

*TEE* transoesophageal echocardiography

TEE revealed LA thrombi in 7 (5.8%) patients, in 5 males and 2 females (71.4% and 28.6%, respectively). In these patients SR was not restored, treatment with OACs was continued. The patients’ age ranged from 44 to 84 years (mean age 64.1 ± 14.9). CHA2DS2-VASc score in these patients ranged from 5 to 7 points. Thrombi were found in 2 (6.1%) of patients with slightly decreased LV function and in 5 (17.9%) of patients with markedly decreased LV function. In patients with decreased LVEF thrombi in LA were found more frequently than in patients with normal and slightly decreased LVEF (17.9 vs 2.2%, *p =* 0.008). Decreased LV systolic function and CHA2DS2-VASc score ≥5 were observed in all male patients. In both female patients LV systolic function was slightly decreased and CHA2DS2-VASc score was 6. Thrombi were detected in 5 (7.0%) patients of warfarin group and in 2 patients (4.1%) of NOACs group. The duration of the preparation period for the scheduled restoration of SR in patients with LA thrombi in warfarin group was longer in comparison with the period in NOACs group (2 – 24 months, mean 11.4 months vs 3 – 8 weeks, mean 6 weeks). A previous history of LA thrombi was found in two male patients, and SR was restored after prolonged anticoagulagion and repeated TEE. After the next AF relapse, TEE revealed LA thrombi again, anticoagulation was prolonged and TEE repeated. One patient did not have LA thrombi, SR was restored and the patient is waiting for LA appendage closure. Another patient still has LA thrombi, and permanent AF was diagnosed. For other 3 patients TEE was repeated after 1 and 3 months, thrombi in LA remained and permanent AF with OAC treatment and rate control was continued. For 2 patients TEE was repeated after 1 month, thrombi in LA resolved and SR was restored, with no TE complications 30 days after cardioversion. In both groups the presence of thrombi did not depend on the grade of LA enlargement.

An interview by phone was performed 30 days after restoration of SR. There were no TE complications in patients four weeks after discharge.

## Discussion

The aim of our study was to assess the diagnostic value of TEE in patients with non-valvular AF anticoagulated according to guidelines, and to establish possible additional indications for TEE and to evaluate incidence of LA thrombi in properly anticoagulated patients in daily clinical practice. In our study this incidence is similar to other studies’ data, in which thrombi in LA were found for 3.6–7.7% patients properly anticoagulated with warfarin before scheduled cardioversion [11, 12]. We found LA thrombi in 7.0% patients in warfarin group and 4.1% in NOACs group, we cannot state that risk of thromboembolism is higher in the warfarin group, because the number of these patients is small and because of mentioned selection bias. It should be noted that the number of patients on warfarin was larger and the duration of preparation for scheduled DCC of patients in whom LA thrombi were revealed lasted longer, in comparison with those in NOACs group, with duration ranging from 2 to 24 months (mean 11 months) vs 3-8 weeks (mean 6 weeks), respectively. The prolonged duration of preparation possibly had an influence on thrombi formation as well.

It is not obligatory to perform TEE for patients properly prepared for DCC by OACs [1, 6]. However, if a physician has any concerns regarding compliance to OAC treatment, or for other reasons that may influence the formation of intracardiac thrombi before scheduled DCC, it is reasonable to perform TEE [6, 17]. In RE-LY study the duration of dabigatran administration before scheduled DCC was ≥3 weeks. In the group of patients who received 110 mg of dabigatran twice daily, TEE was performed for 25.5%. LA thrombi were detected in 1.8% of these patients. In the group of patients who received 150 mg of dabigatran twice daily, TEE was performed in 24.1% of the cases and LA thrombi were detected in 1.2% of the patients [18]. In ARISTOTLE study, for patients prepared for scheduled DCC with apixaban, TEE was performed for 36.6% of the patients and no LA thrombi were found. However, the treatment duration prior to restoration of SR was long (251 ± 248 days) [19]. The question whether the treatment with NOACs lasting from 3 to 4 weeks is sufficient to prepare the patient for scheduled DCC remains an object for discussion. The study of dabigatran demonstrated that preparation for scheduled DCC for 4 weeks is safe [20] but the problem of compliance with the treatment still remains. The half-life of NOACs (about 12 hours) is markedly shorter in comparison with warfarin; therefore, missing of a dose at the appropriate time results in the rapid decrease of anticoagulation action. The patients comply with the treatment of NOACs better than these on warfarin, but the accuracy of use of preparations is not clear. During a one-year period of observation, 47.5% of the patients receiving NOACs compared to 40.2% of the patients on warfarin, complied with the treatment for 80% of observation period (p < 0.001) [10]. In order to assure appropriate use of the drug, more strict control is required. This is why additional TEE is being performed, even in patients properly prepared with OACs for planned SR restoration [1, 6, 9]. We did not find any relationship between the decision to perform TEE and the estimated CHA2DS2-VASc score. The age of the patient was not considered as a decisive factor to carry out TEE. Approximately half of the patients (49.2%) who underwent TEE were younger than 65 years.

In our study, more than a half of the patients (55.2%) were obese (BMI ≥30 kg/m^2^). Obesity increases the risk of development of a new AF, the incidence of AF relapses and the risk of development of permanent AF. In obese patients with AF, the decrease of the weight reliably decreases the incidence of AF relapses and total duration of the episodes [21]; on the other hand, there are controversial data if obese patients with AF have an increased risk of TE events. According to our data, this factor had no influence on the incidence of AF relapses, the decision to perform TEE, or the detection of heart chamber thrombi.

Male and elderly patients received NOACs more frequently in our study. Warfarin was more frequently administered to the patients with a higher risk of TE complications and history of stroke or TIA. The incidence of NOACs administration was higher for patients with normal systolic LV function, without LA enlargement, while patients with decreased LVEF or LA enlargement received warfarin more frequently.

The majority of patients (73.9%; *p =* 0.087) with a high risk of bleeding (HAS-BLED ≥3) used warfarin. For patients who had a high TE risk (CHA2DS2-VASc ≥6) only warfarin was administered. Warfarin was more frequently administered to the patients suffering from CAD, possibly because of ESC 2015 Guidelines recommending exclusive prescription of warfarin as a part of triple anticoagulation therapy after acute coronary syndromes or scheduled percutaneous transluminal coronary angioplasty. TEE was performed in NOACs group more frequently, in comparison with the warfarin group (38.9 vs 23.2%).

In the study performed by Zylla et al. [17] 643 patients with non-valvular AF and prepared for scheduled DCC by VKA and NOACs were evaluated. TEE was performed for all study subjects. The mean CHA2DS2-VASc score was 4. LA thrombi were detected in 10.6% of the patients and the incidence of thrombi was higher in the warfarin group (17.8 vs 3.9%). In patients for whom TEE revealed thrombi they had a higher CHA2DS2-VASc score (4–5 points in 49%), larger LA or suffered from LV systolic dysfunction. The incidence of thrombi detected was higher in patients treated by VKA with intra-cardiac devices (pacemakers, defibrillators), suffering from CAD. The patients in NOACs group were younger, had a lower CHA2DS2-VASc score, less dilated LA, and better LV systolic function; the incidence of NOACs administration was lower for patients with intra-cardiac devices implanted. The data of our study are quite similar. LA thrombi were revealed in 7 (5.8%) of patients properly prepared using OACs for SR restoration. We found out that the mean CHA2DS2-VASc score was 3.5, but the sample of patients with 4 points was the largest (24.5%). In the case when the CHA2DS2-VASc score was ≥6, only warfarin was administered. The patients on NOACs were younger in our study and they also had less concomitant diseases. However, in patients >75 years old NOACs were administered more frequently, possibly because of difficulties in monitoring INR while administering warfarin, and INR lability.

Similarly, to the data of Zylla et al. [17], in our study the incidence of LA thrombi was higher in warfarin group. Patients in whom intracardiac thrombi were revealed had a higher CHA2DS2-VASc score, and decreased LV systolic function. In two patients who had history of LA thrombi TEE revealed newly formed ones. Both of these patients were on warfarin and their CHA2DS2-VASc score was 6.

The risk of TE complications in the event of markedly decreased LV systolic function (LV EF < 35%) in females is two times higher, in comparison with the risk in males [22]. However, the incidence of thrombi in males, according to our data, is two times higher.

In our study, an interview by phone 30 days after restoration of SR showed no TE complications.

This retrospective study was based on everyday clinical practice and demonstrated that even for patients properly prepared for SR restoration, TEE was performed quite extensively. The risk of thrombi formation, when the patient is prepared by OACs for restoration of SR and adequate anticoagulation is achieved, is low but still remains. Therefore, in all cases the risk for a patient should be evaluated individually.

### Limitations

The number of patients in whom LA thrombi have been identified is small (n = 7). It is necessary to perform larger studies of properly anticoagulated patients to make firm conclusions.

## Conclusions

The risk of LA thrombi in patients who are prepared for scheduled SR restoration according to guidelines is low. The higher risk of cardiac thrombi was present in patients with decreased LVEF (≤40%), CHA2DS2-VASc ≥5. A larger number of patients is required, in order to assess more precise TEE indications for properly anticoagulated patients prior to scheduled cardioversion.

## Abbreviations

AF: Atrial fibrillation
AH: Arterial hypertension
BMI: Body mass index
CAD: Coronary artery disease
DCC: Direct current cardioversion
DM: Diabetes mellitus
HF: Heart failure
INR: International normalized ratio
LA: Left atrium
LAA: Left atrial appendage
LV: Left ventricle
LVEF: Left ventricular ejection fraction
LVH: Left ventricular hypertrophy
NOAC: Non-vitamin K antagonist oral anticoagulants
OACs: Oral anticoagulants
SD: Standard deviation
SR: Sinus rhythm
TE: Thromboembolic
TEE: Transoesophageal echocardiogram
TIA: Transient ischemic attack
VKA: Vitamin K antagonists
